# Quantitative evaluation of Zindagii Shoista (Living with Dignity) intervention to prevent violence against women in Tajikistan

**DOI:** 10.1080/16549716.2022.2122994

**Published:** 2022-11-28

**Authors:** Subhiya Mastonshoeva, Shahribonu Shonasimova, Parvina Gulyamova, Rachel Jewkes, Nwabisa Shai, Esnat Chirwa, Henri Myrttinen

**Affiliations:** a International Alert, Dushanbe, Tajikistan; b CESVI, Dushanbe, Tajikistan; c Office of the Executive Scientist, South African Medical Research Council, Pretoria, South Africa; d School of Public Health, Faculty of Health Sciences, University of the Witwatersrand, Johannesburg, South Africa; e Gender & Health Research Unit, South African Medical Research Council, Pretoria, South Africa; f Gender Associations International Consulting, Berlin, Germany

**Keywords:** Violence against women and girls, intimate partner violence, domestic violence, women economic empowerment

## Abstract

**Background:**

Violence against women and girls (VAWG) is a major problem in Tajikistan, driven by conservative gender norms, the culturally ascribed position of young women, and poverty.

**Objective:**

We evaluated Zindagii Shoista (Living with Dignity), an intervention developed with the aim of reducing VAWG through a combination of gender norm change, communication skills, and income-generating activities (IGA) over a period of 30 months.

**Methods:**

The evaluation used a mixed-methods approach, combining quantitative and qualitative data collection. Eighty families from four villages were enrolled in the intervention and surveyed at baseline and on three subsequent occasions. From these families, 134 women and 102 men were interviewed at baseline, 153 women and 89 men 8 months later, 153 women and 93 men 15 months later, and 143 women and 82 men, 30 months after the baseline. Generalised random effects regression models were used to assess the trend in proportions or mean score over time.

**Results:**

Over the 30 months, the proportion of women and men earning in the past month rose from 17.9% to 56.6% and 44.1% to 72%, respectively. Women and men’s gender attitudes became significantly less patriarchal, and they reported less harmful gender norms in the community. Women and men reported less male controlling behaviour and greater woman involvement in decision-making. Women’s reports of experience of emotional, physical, and sexual IPV significantly reduced. Depressive symptoms and suicidal thoughts reduced significantly for men and women, and self-rated health improved.

**Conclusions:**

The quantitative findings are confirmed by the findings of the qualitative research and monitoring data. They demonstrate that Zindagii Shoista is a very promising intervention for strengthening gender relations, reducing IPV, and improving mental health and socio-economic circumstances for younger married women and their families in Tajikistan.

## Background

In Tajikistan, in the heart of Central Asia, violence against women and girls (VAWG) is a substantial problem, affecting at least half of women in their lifetime [1]. Levels of emotional abuse are particularly high, with a household survey in Khatlon Region reporting that it was experienced by 37.3% of women in the previous year, and 40.3% of women had ever experienced marital rape [2]. VAWG is driven by the patriarchal social norms, cultural traditions around co-habitation with the husband’s in-laws on marriage, and the privileging of elders over younger people, as well as severe poverty. Women generally marry young, moving into their husband’s family home, and are vulnerable to violence from their husbands, as well as members of his family [3–8]. Acceptance of violence and controlling behaviour is high and linked to norms, supported by men and women alike, around the importance of protecting family honour and linking this to younger women’s behaviour [3,8]. Tajikistan is a low-income country, with the greatest income generating opportunities for men lying in labour migration to Russia. Usually, when men migrate, their wives are left with their in-laws.

Recognising the nexus of patriarchy and poverty in driving VAWG and constraining women’s power and possibilities, researchers and implementers have sought to combine economic empowerment and gender transformative interventions, with different degrees of success in impacting VAWG [9]. There have been good examples of interventions that have effectively combined a gender transformative elements with microfinance [10], or cash transfers [11], but there has been little research with interventions that establish income-generating projects. Mostly, the economic intervention has targeted women, but a notable exception of Stepping Stones and Creating Futures, used in South Africa, which has had considerable success through also focusing on men [12,13]. *Zindagii Shoista* (Living with Dignity) was developed to prevent VAWG in Tajikistan, combining an attitudinal and gender norm change component with life skills and income-generating activities, drawing on the experiences of Stepping Stones and Creating Futures [14,15]. To our knowledge, it is the first intervention to be developed in Central Asia that seeks to prevent VAWG through working across two generations within the family and it is the first VAWG prevention intervention developed specifically for the Tajikistan cultural context. In this paper, we present the findings of research conducted with the aim of showing proof of the concept, particularly whether the intervention model combining behavioural change and income-generating activities showed promise in reducing VAWG in the target families and what other short- to medium-term impacts of the intervention might be attributed to the intervention.

## Methods

### Setting

The Zindagii Shoista intervention was piloted in four villages, two in the northern district of Penjikent and two in the southern district of Jomi, as part of the global DFID-funded ‘What Works to Prevent VAWG?’ programme. The Penjikent villages were in the northern mountainous region of the country with a predominantly Uzbek population, and the Jomi villages were on the southern plains region with a predominantly ethnic Tajik population. The villages had a comparatively low standard of living and limited economic opportunities, with a high level of external migration, high level of withdrawal of girls from school, early marriage, and divorce. The logic for choosing the four villages was to allow for a comparison between two areas with different regional dynamics (northern mountainous region vs. southern plains region) as well as different ethnic compositions (predominantly ethnic Uzbek and Tajik, respectively), and by comparing for proximity to an urban centre.

### Participants

We selected 20 families from each village for the pilot and intended to involve two men and two women per family, across two generations. The sample size was based on feasibility. Families were selected based on vulnerability criteria developed jointly by Alert, Cesvi, and local NGO partners and based on the formative research findings. The families were known locally to have difficulties, including younger women experiencing intimate partner violence (IPV) and violence from in-laws.

### The Zindagii Shoista intervention

**Figure 1.**
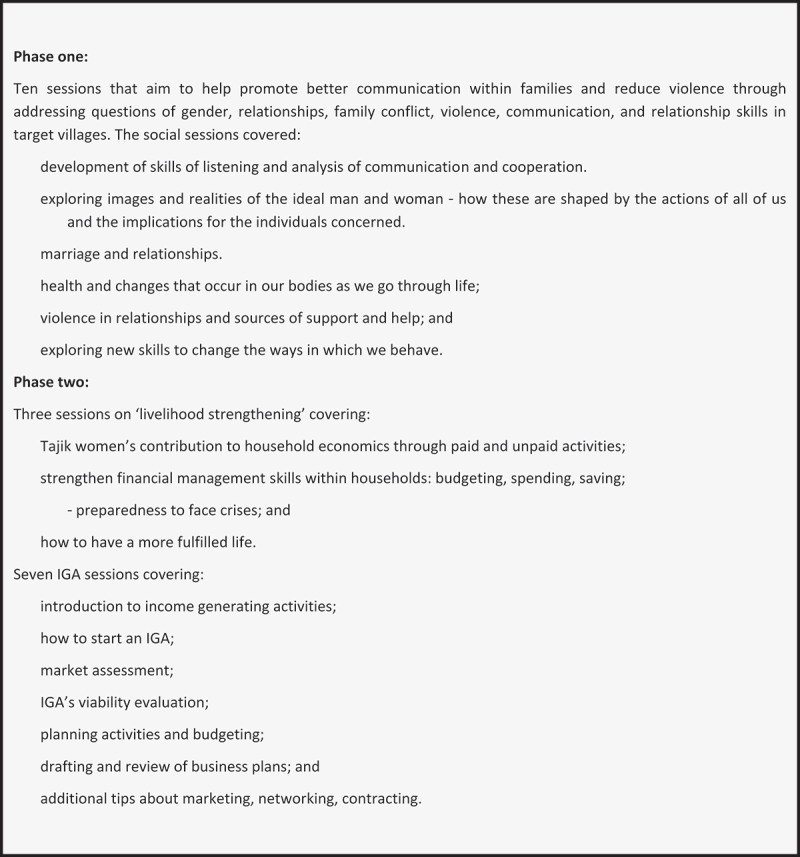
Outline of Zindagii Shoista.

The Zindagii Shoista intervention was developed for use with multi-generational families and combines critical reflection on gender relations, the impact and origins of violence, social value of men and women in the home, and communication skills, and seeks to apply these skills to enhance family relationships (Figure 1). It combines these with a fully supported programme to enable the development of a small-family-led enterprise in which young women have an active role, with business skills, small start-up funds, and technical assistance. It is fully described in two intervention manuals, and its development is presented elsewhere [14–16]. The gender empowerment and communication skills component of 13 sessions was developed from elements of the South African adaptation of Stepping Stones [17] and the livelihood strengthening intervention Creating Futures [18], but the business development component of seven sessions was developed mostly anew, although, based on the business development model of our international non-governmental organisation partner Cesvi [18]. Whilst other gender empowerment and economic interventions to prevent IPV have been evaluated elsewhere when delivered to women and men, the approach chosen for Tajikistan was based on the extended family rather than a husband/wife dyad (cf. Falkingham and Baschieri 2009 [19]) and had not been previously tested [9].

The programme is based on participatory learning approaches, affirming participants’ knowledge, and enabling them to discuss and decide things for themselves. They were conducted by trained facilitators from the local NGO partners. Facilitators were selected based on their understanding of the local context, language, facilitation skills, and respect within communities locally, enabling them to gain access to families and facilitate discussions on sensitive topics. Business and technical assistants were chosen as individuals with previous experience of establishing IGAs, household money management, and understanding of the business environment. Facilitators for both components were first given a chance to experience the sessions as participants followed by teaching the sessions to their colleagues to build expertise. Additional 2-day gender-sensitisation training was conducted, mainly to build the capacity of male facilitators and business assistants implementing the two components of the project to ensure understanding of the gender-transformative aims of the intervention and how to implement this in a gender-sensitive manner.

The programme was delivered to age- and gender-divided peer groups of 15–20 people in each village: older men, older women, young men, and young women. But all groups came together periodically for joint discussions to share what they have been learning and discussing. The sessions were approximately 3 hours long and were delivered over 20 weeks. The sessions were given once to each group at the beginning of the project. The families afterwards were accompanied by the facilitators and business assistants throughout the project to help with the development and running of IGAs as well as monitoring the changes in power dynamics and relationships in the family. The average attendance rate in both economic and social sessions was approximately 72%. In all, 80 income-generating activities (IGAs) were established by the families and the businesses included baking, tailoring, beekeeping, cattle breeding, renting out plastic tables/kitchen utensils for events, poultry, and greenhouses.

### Evaluation design and data collection

The pilot was evaluated using mixed methods, with careful monitoring of the intervention by observing sessions. In this paper, we present the findings of the quantitative component which was a modified interrupted time series with four data points. At baseline, in September 2016, 134 women and 102 men participating in the intervention were interviewed with a standard questionnaire. We repeated interviews 8, 15, and 30 months later, interviewing 153 women and 89 men at 8 months, 156 women and 93 men at 15 months (end point of the intervention), and 143 women and 82 men at 30 months (15 months after the end of the intervention). The number of men interviewed mainly fluctuated due to labour migration, mainly to Russia. The number of women was higher at subsequent interview rounds than baseline due to the latter being coincided during the cotton harvest season of the target districts, in which women are particularly engaged. The fieldwork strategy was adjusted after baseline to improve retention of the sample.

The questionnaire covered the socio-economic situation of respondents, family relations, gender attitudes, experiences of violence, physical and mental health, as well as hopes for the future. Complementary questionnaires were developed for women and men, with the focus of the violence questions on male perpetrated intimate partner violence and violence from the husband’s mother. Details of the items are presented in Table 1. The questionnaires were translated into Tajik and Uzbek languages. The interviews were conducted by male and female interviewers conducting same-sex interviews.

**Table 1.** Measures used in the survey instrument[20–28].

Construct	Measure	Typical items	Origin
Severe food insecurity	Household members experiencing a lack of food in the previous 4 weeks (3 items)	In the past 4 weeks, how often was there no food to eat of any kind in your house because of a lack of money? Severely food insecure was defined as a reply of often to 1 or more of the questions	Coates et al [24] Household Food Insecurity Access Scale (HFIAS)
Income-seeking effort scale	Effort trying to get a job or earn money by selling or making things in the last 3 months (7 items)	Typical item: how often have you … Searched newspapers for jobs	Barker G, et al [25]. IMAGES study questionnaire.
Stress due to not having work or enough money	4-item scale with questions about stress related to current work situation	‘I am frequently stressed or depressed because of’ not having enough work or enough income	Barker G, et al [25]. IMAGES study
Unemployment shame	4-item measure of shame and despondency	Typical item: I sometimes feel ashamed to face my family because I am out of work	Barker G, et al [25]. IMAGES study
Depression	CES-D scale (used as a continuous variable)	Typical item: During the past week, I felt I could not cheer myself up even with the help of family and friends Scored high = more depression	Radloff [26]
Alcohol	Women: 3 items on husbands’ drinking; Men: 3 items on their drinking	Does he drink? How often? In past 12 m, how often has she seen him drunk? Men asked: Ever drunk? Drunk in past 12 m? How often in past 12 m?	
Hope	Scale of 6 items	Typical item: I can think of many ways to get out of a difficult situation	
Disability	Disability due to a health problem or injury (6 items)	Typical item: Do you have difficulty walking or climbing steps?	Washington Group Scale
Suicidal thoughts	Single item on suicidality in last 4 weeks	In the past four weeks, has the thought of ending your life been in your mind?	
Self-rated health	Single item with five response categories	In general, would you describe your overall health as excellent, good, fair, poor or very poor?	
Individual gender attitudes	22 items with Likert responses: strongly agree, agree, disagree, and strongly disagree	Typical item: I think that the daughters-in-law in my family must always obey their mother-in-law	Jewkes et al [29], lightly adapted for the Tajik context after formative research
Community gender attitudes (social norms)	22 items with Likert responses: strongly agree, agree, disagree, and strongly disagree	Typical item: In this community, many people think that if a wife does something wrong, her husband has the right to punish her	Jewkes et al [29], lightly adapted for the Tajik context after formative research
Wife’s relationship with her husband	4 items, statements with Likert responses	Typical item: My husband is a kind person	Based on Jewkes et al [20] adapted for the Tajik context after formative research
Man’s relationship with his wife	5 items, statements with Likert responses	Typical item: My wife does everything she can to support me	
Women’s mother-in-law cruelty	3 items summed: statements with Likert responses	Typical item: My mother-in-law is very strict and controlling	
Women’s mother-in-law kind	3 items summed: statements with Likert responses	Typical item: My mother-in-law is a kind person	
Man’s assessment of his mother’s cruelty	2 items summed: high = more cruel	Typical item: My mother can frighten me	
Man’s assessment of his mother’s kindness	3 items summed: high = more kind	Typical item: My mother does everything she can to support me	
Women’s involvement in decision-making	5 items scored high = more involvement	In the last three months, how often have your views been listened to on problems which your husband or family faces?	
Relationship control measure	8 items, statements with Likert responses	A typical item is he won’t let me spend money on things for myself	Jewkes et al [21], adapted for Tajikistan
Frequency of quarrelling	Single item	In your relationship with your husband, how often would you say that you quarrelled?	Based on Jewkes [21]
Physical IPV	5 items (exposure = experience of any act 1 or more times)	Typical item for men: In the past 12 months, how many times have you hit her with a fist or with something else which could hurt her?	Garcia-Moreno et al [22]; Fulu et al [23]
Sexual IPV	3 items (exposure = experience of any act 1 or more times)	Typical item for women: In the past 12 months, how many times has your husband used threats or intimidation to get you to have sex when you did not want to?	Garcia-Moreno et al [22]; Fulu et al [23]
Emotional IPV	11 items (exposure = experience of any act 1 or more times)	Typical item for women: In the past 12 months how, many times has your husband insulted you or made you feel bad about yourself?	Garcia-Moreno et al [22]; Fulu et al [23]

### Research implementation and ethics

The local NGO partners secured approval from local formal and informal leaders for the study. Access to villages and written permission to work in the villages was obtained from the local governments of the targeted district after several meetings with relevant local governmental officials to explain the details of the project and the research. The local partners also assisted the research team with the recruitment of respondents, administrative arrangements for the survey, and offering light refreshments to the respondents. The researchers ensured cohort retention by engagement with the families through the intervention and intermittently after the intervention support had finished.

The research protocol was reviewed and approved by the SAMRC’s Human Research Ethics Committee, and written informed consent was obtained from the study participants for the survey in all target villages. The research was informed by the WHO guidelines on research on violence against women [29]. Interviews were conducted by trained interviewers in private and were interrupted if participants expressed distress. Counselling after the interviews for any participants requesting it was available from the NGO partners, but we do not know if services were used.

### Analysis of the data

Analysis was by intention to treat; thus, we included all participants enrolled into the intervention at baseline irrespective of their attendance in the intervention training programme. Descriptive statistics such as frequencies, percentages, means, and standard deviations were used to summarise socio-demographic characteristics of participants. We used percentages to summarise categorical or dichotomous outcomes at each data collection point. For scales such as relationship control, depression, gender attitudes, and decision-making scales, we derived a summative score from the item responses and presented mean scores as summary statistics at each time point. The level of missing data varied between items and scales. It was higher at baseline (up to 17%) than for the third and fourth waves (less than 10%). There were no significant differences in the proportion of missing item data between male and female participants. About 20% of participants were lost to follow-up (i.e were available at baseline but not available at either third or fourth data collection waves), with similar distribution between male and female participants. We performed missing data analysis to assess if there were any baseline socio-demographic factors that were associated with loss to follow-up. We defined loss to follow-up as not being available for interviews at 15th and 30th months (third and fourth data waves). We found no association between loss to follow-up and socio-demographic factors or with any of the study outcomes. In order to maintain the sample size, we used multiple imputation to deal with missing data in the item responses for continuous study outcomes at each data collection. Generalised random effects regression models were used to assess the trend in proportions or mean score over time, with each participant as a random component (cluster) in the model. Study time points were entered as the main exposure and were used to determine trends in each outcome measure over time. All random effects models were adjusted for the age of the participants and all analyses were done in Stata 14. We analysed the data for male and female participants separately.

## Results

### Social and demographic characteristics

The ages of the respondents ranged from 16 to 65 years old, and the women were a little younger than the men and most were currently married (Table 2). The proportion of ethnic Tajiks and Uzbeks was almost equal. Men were significantly more likely to have studied beyond secondary school than the women (59.8% vs. 27.6%). Women reported, much more commonly than men, living with their spouse’s family (39.0% vs. 5.9%), and 32.2% of women and 24.7% of men were living in a nuclear family (spouse and children). More women were reported to be in a polygamous marriage than men (14.9% vs. 4.9%). Labour migration patterns were heavily gendered, with 49.0% of men and only 8.2% of women having ever migrated for work.

**Table 2.** Social and demographic characteristics of participants at baseline.

	Women (N = 134)		Men (N = 102)	
	N	%	N	%
**Age group** (women: n = 131; men: n = 100)				
16–24 yrs	22.0	16.8	13.0	13.0
25–34 yrs	47.0	35.9	30.0	30.0
35–44 yrs	25.0	19.1	18.0	18.0
45–54 yrs	18.0	13.7	10.0	10.0
≥55 yrs	19.0	14.5	29.0	29.0
**Ethnicity**				
Tajik	67.0	50.0	46.0	45.1
Uzbek	62.0	46.3	50.0	49.0
Other	5.0	3.7	6.0	5.9
**Education level** (men n = 99)				
None	6.0	4.5	2.0	2.0
Primary	11.0	8.2	5.0	5.1
Secondary	78.0	58.2	30.0	30.3
Above secondary	39.0	29.1	62.0	62.6
**Currently married**	92.0	68.7	78.0	76.5
**Married to a relative**	29.0	21.6	10.0	9.8
**Living arrangements**				
Lives with husband/wife and children	38.0	32.2	21.0	24.7
With husband’s family (women) or wife’s family (men)	46.0	39.0	5.0	5.9
With husband/wife and own family	6.0	5.1	39.0	45.9
Living with children without a spouse	13.0	11.0	14.0	16.5
Living with natal family (no spouse or children)	12.0	10.2	6.0	7.1
Living alone	3.0	2.5	0.0	0.0
**In polygamous marriage**	20.0	14.9	5.0	4.9
**Ever migrated for work**	11.0	8.2	50.0	49.0
**Average number of children (SD)**	4.0	1.9	5.0	4.9

### Socio-economic status

At baseline, the socio-economic status of the participating families was low. Over the 3 months prior to the survey, less than a quarter of women (23.9%) and only just over half of men (54.9%) had earned money. Only 6.7% of women and 22.6% of men had any savings. Most men and women perceived it would be difficult to raise 500 Somonis (at the time, US$ 63.5) in case of an emergency. Most women and men said that they had borrowed money or food in the previous month due to not having enough (61.9% women and 54.8% men) and disclosed severe food insecurity (55.7% of women and 33.8% of men). Their socio-economic position caused them considerable stress and shame.

Over the period of 30 months, there were significant positive changes in the economic situation of the families, and most of these changes were reported as starting in the first year (Table 3). Substantial increases were seen in the proportion of women and men with earnings and savings in past month, and the proportion with overall savings. At 30 months, 56.6% of women and 72.0% of men had earned money in the past month, and 40.6% of women and 51.2% of men had savings. There was some reduction in the proportion of women with earnings and savings from 15 months to 30 months, but the proportion earning was still more than three times higher than that at baseline. The proportion of men with earnings and savings was sustained between 15 and 30 months. The positive changes in socio-economic indicators were mirrored by reports of change from the qualitative research and monitoring data [30].

**Table 3.** Trends over time in socio-economic status of participants.

	Baseline	8 m	15 m	30 m		
	mean/%	mean/%	mean/%	mean/%	Coefficient (95% CI)	P value
**Women**	**n = 134**	**n = 153**	**n = 153**	**n = 143**		
Any earnings in past month (%)	17.9	63.4	78.9	56.6	0.03(0.01,0.05)	0.001
Any savings in past month (%)	6.7	43.8	55.1	40.6	0.03(0.01,0.04)	0.002
Any overall savings (%)	6.7	59.5	62.2	53.9	0.04(0.02,0.05)	<0.001
Done something to earn money in last 3 months (%)	23.9	66.0	79.5	55.2	0.02(−0.001,0.03)	0.065
Difficult to find 500 Somonis in an emergency (%)	87.3	13.7	10.9	22.4	−0.08(−0.10, −0.06)	<0.001
Borrowed food or money in past month because there was not enough (%)	61.9	39.9	35.9	34.3	−0.03(−0.05, −0.02)	<0.001
Severely food insecure v. moderate or none (%)	44.0	19.7	19.4	2.8	−0.14(−0.17, −0.10)	<0.001
Effort to get a job or earning money by selling or making things score (high = more effort)	10.6	9.9	11.5	10.7	0.02(0.004, 0.05)	0.022
Stress due to not having work or enough money (high = more stress)	10.9	10.8	9.4	8.5	−0.08(−0.10, −0.07)	<0.001
Shame and despondency due to lack of work (high = more shame)	10.7	8.4	7.5	6.5	−0.11(−0.13, −0.10)	<0.001
**Men**	**n = 102**	**n = 89**	**n = 93**	**n = 82**		
Any earnings in past month (%)	44.1	69.7	71.0	72.0	0.05(0.02, 0.07)	0.001
Any savings in past month (%)	14.7	43.8	44.1	51.2	0.05(0.03, 0.08)	<0.001
Any overall savings (%)	22.6	69.7	66.7	72.0	0.07(0.04, 0.10)	<0.001
Done something to earn money in last 3 months (%)	54.9	68.5	77.4	73.3	0.03(−0.003, 0.05)	0.083
Difficult to find 500 Somonis in an emergency (%)	87.3	86.5	85.0	70.7	−0.06(−0.09, −0.03)	<0.001
Borrowed food or money in past month because there was not enough (%)	57.8	33.7	10.8	13.4	−0.12(−0.16, −0.08)	<0.001
Severely food insecure v. moderate or none (%)	33.8	4.6	0.0	0.0	−1.55(−2.38, −0.71)	<0.001
Effort to get a job or earning money by selling or making things score (high = more effort)	8.4	8.5	9.6	9.0	0.02(−0.01, 0.04)	0.169
Stress due to not having work or enough money (high = more stress)	10.9	10.0	8.7	8.9	−0.04(−0.07, −0.02)	0.001
Shame and despondency due to lack of work (high = more shame)	11.5	9.3	8.1	8.1	−0.06(−0.08, −0.03)	<0.001

Only two of the trends in socio-economic indicators were not statistically significant. One was the indicator of men’s efforts to get a job or earn, which showed improvement over the period from baseline to 30 months, although the highest score was at 15 months. The change in this measure was significant for women, although the actual scores did not show a convincing change. The other measure was the proportion of women and men doing something to earn money in the past 3 months, which increased twofold for women between baseline and 30 months (from 23.9% to 55.2%) and by one-third for men (from 54.9% to 73.3%), albeit with some back sliding from the 15 months highs at the end of the intervention, but these changes were not statistically significant.

### Mental health outcomes

At baseline, mental health and general health in the participant families were poor. The mean score of the CES-D depression scale for women and men were 28.5 and 17.9, respectively; both were above the commonly used cut points for substantial depression symptomatology (16+) (Table 4). In the previous 4 weeks, 12.9% of women and 4.9% of men had had suicidal thoughts. A disability due to a health problem was reported by 9.4% of women and 8.4% of men. Less than three-quarters of women and men (73.9% and 68.6%) described their health as fair, good, or excellent at baseline; the others perceiving it to be poor or very poor.

**Table 4.** Trends in mental health outcomes.

	Baseline	8 m	15 m			
	%/mean	%/mean	%/mean	30 m	Coefficient (95% CI)	P value
**Women**	**n = 134**	**n = 153**	**n = 153**	**n = 143**		
Depression score (low = good)	28.5	17.4	15.1	13.6	−0.36(−0.42, −0.30)	<0.001
Hope scale (high = more hopeful)	17.1	18.8	18.3	18.6	0.02(0.01, 0.04)	0.009
Disability score (high = more disability)	9.5	7.0	7.9	7.6	−0.03(−0.10, −0.02)	<0.001
Suicidal thoughts in last 4 weeks (%)	12.5	1.3	0.6	2.1	−0.06(−0.1, −0.02)	0.001
Fair, good, or excellent general health (%)	73.9	91.5	93.6	91.6	0.04(0.01, 0.07)	0.006
**Men**	**n = 102**	**n = 89**	**n = 93**	**n = 82**		
Depression score (low = good)	17.9	10.5	6.9	8.4	−0.25(−0.3, −0.19)	<0.001
Hope scale (high = more hopeful)	16.9	18.0	18.4	18.4	0.06(0.04, 0.08)	<0.001
Disability score (high = more disability)	8.4	6.9	6.8	6.6	−0.04(−0.05, −0.03)	<0.001
Suicidal thoughts in last 4 weeks (%)	4.9	1.1	0.0	0.0	−0.47(−0.81, −0.13)	0.007
Fair, good, or excellent general health (%)	68.6	96.6	97.9	92.7	0.07(0.02, 0.12)	0.003

Over the time of the research, there was a significant and sustained decrease in depression among both women and men and a significant positive change in hope (Table 5). The 30-month mean depression scores were well within the non-depressed range for both women and men (13.6 and 8.4). The high proportion of women with suicidal thoughts at baseline was significantly reduced with only 2.1% of women and no men reporting suicidal thoughts in the past month at 30 months. The disability scores for men and women also significantly reduced. Self-rated health also improved, with 91.6% of women and 92.7% of men rating themselves as being in fair, good, or excellent health at the end line, a substantial improvement from baseline.

**Table 5.** Trends over time in gender attitudes and relationships.

	Baseline	8 m	15 m			
	%/mean	%/mean	%/mean	30 m	Coefficient (95% CI)	P value
**Women**	**n = 134**	**n = 153**	**n = 153**	**n = 143**		
Individual gender attitudes (high = patriarchal)	49.8	49.0	46.1	45.2	−0.16(−0.19, −0.12)	<0.001
Community attitudes (high = patriarchal)	53.6	52.1	48.0	48.2	−0.18(−0.23, −0.14)	<0.001
Woman’s relationship with husband (high = better relationship) (4 items)	10.4	9.8	9.4	10.3	0(−0.01, 0.01)	0.802
Woman’s mother-in-law cruel (high = more cruel) (4 items)*	9.9	8.7	8.0	9.0	−0.01(−0.04, 0.01)	0.271
Woman’s mother-in-law kind (high = more kind) (3 items)*	7.4	8.3	8.8	7.6	−0.01(−0.03, 0.0)	0.117
Woman involvement in decision-making (high = more involvement)	7.3	9.0	10.0	10.1	0.07(0.05, 0.01)	<0.001
Women controlled by husband (high = more control)	20.8	18.6	19.3	18.7	−0.03(−0.05, 0.0)	0.024
Quarrelling frequently (%)	12.7	4.6	4.5	5.6	−0.02(−0.06, 0.01)	0.232
Husband drinks alcohol (%)	27.6	17.7	12.2	16.1	−0.02(−0.05, 0.0)	0.088
**Men**	**n = 102**	**n = 89**	**n = 93**	**n = 82**		
Individual gender attitudes (high = patriarchal)	52.8	46.3	41.2	42.9	−0.27(−0.34, −0.19)	<0.001
Community attitudes (high = patriarchal)	53.7	52.7	46.5	45.7	−0.27(−0.32, −0.22)	<0.001
Man’s relationship with wife (high = better relationship) (5 items)	15.3	16.1	16.9	16.2	0.01(−0.01, 0.03)	0.186
Man’s mother cruel (high = more cruel) (2 items)*	4.4	3.4	2.5	3.2	−0.03(−0.05, −0.01)	0.006
Man’s mother kind (high = more kind) 3 items*	9.7	9.5	9.6	9.5	0(−0.03, 0.02)	0.811
Woman involvement in decision-making (high = more involvement)	8.1	7.6	8.7	9.8	0.07(0.05, 0.10)	<0.001
Women controlled by husband (high = more control)	19.5	17.8	16.3	16.1	−0.08(−0.11, −0.05)	<0.001
Quarrelling frequently (%)	5.9	3.4	0.0	0.0	−0.03(−0.12, 0.06)	0.468
Alcohol use in past year (%)	34.3	41.6	23.7	14.6	−0.08(−0.12, −0.03)	0.001

*Analysis of married women and men aged <40 years.

### Gender attitudes and relationships

Across the four-time points, women and men’s gender attitudes became significantly more equitable, with notable change in the views of men. In parallel with this, women and men perceived changes in social norms in the community, perceiving these also to become more equitable (Table 5). Women and men both reported over time significantly greater involvement of women in decision-making, and women and men reported significantly less controlling behaviour by husbands towards their wives. Men reported significantly less alcohol use over the time, and women also reported their husbands drinking less, although this indicator was not statistically significant despite the magnitude of change being great. Both men and women reported substantial reductions in their having frequent quarrels, this halved for women and reduced to zero reports for men, but the differences were not statistically significant.

The impact on some domains of household relations was less clear. Men reported that their relationship with their wife had improved, although there was some backsliding from 15 to 30 months, and the trend was not statistically significant. Women, however, did not report any improvement in their relationship with their husband. Women reported a reduction in cruelty from their mother-in-law and an increase in perceptions of her kindness, particularly between baseline and 15 months, but there was some backsliding at 30 months, although still showing overall improvement. The trend was not statistically significant. Men, however, reported that their mother’s relationship with their wives was significantly less cruel, but they did not report it to be more kind.

### IPV perpetration or experience

The proportion of women reporting experiencing almost all forms of IPV decreased significantly across the time points, and was sustained to the 30 months’ interviews, which was 15 months after the end of the project support for the intervention (Table 6). Men’s reports of perpetration were at all points lower than reports of women but also decreased across the project, with the reductions sustained after the end of the intervention. The one exception was women’s reports of sexual IPV, which declined very greatly from baseline to 15 months, but at 30 months, were close to the levels reported at 8 months. This was not a significant decline, but it was a reduction of 50% from baseline.

**Table 6.** Trends in IPV experience and perpetration of currently married women and men.

	Baseline	8 m	15 m	30 m		
	%	%	%	%	Coefficient (95% CI)	P value
**Women**	**n = 107**	**n = 131**	**n = 133**	**n = 121**		
Physical IPV in past 12 months	44.9	20.6	16.5	15.7	−0.05(−0.08, −0.03)	<0.001
Sexual IPV in past 12 months	29.0	15.3	3.8	14.9	−0.02(−0.05, 0.0)	0.089
Physical or sexual IPV in past 12 months	47.7	29.8	18.1	24.8	−0.03(−0.05, −0.01)	0.008
Emotional IPV in past 12 months	64.5	43.5	32.3	28.1	−0.06(−0.08, −0.03)	<0.001
Severe sexual or physical IPV (more than 2 acts) in past 12 months	42.1	22.9	14.3	16.5	−0.05(−0.07, −0.02)	0.001
Emotional, sexual, or physical IPV in past 12 months	66.4	49.6	33.1	37.2	−0.04(−0.06, −0.02)	<0.001
**Men**	**n = 87**	**n = 85**	**n = 85**	**n = 87**		
Physical IPV in past 12 months	28.7	9.4	0.0	2.6	−0.21(−0.33, −0.1)	<0.001
Sexual IPV in past 12 months	21.8	10.6	0.0	1.3	−0.22(−0.35, −0.09)	0.001
Physical or sexual IPV in past 12 months	33.3	15.3	0.0	3.9	−0.16(−0.24, −0.08)	<0.001
Emotional IPV in past 12 months	44.8	21.2	4.6	6.4	−0.13(−0.18, −0.07)	<0.001
Severe sexual or physical IPV (more than 2 acts) in past 12 months	28.7	9.4	0.0	2.6	−0.20(−0.32, −0.09)	<0.001
Emotional, sexual, or physical IPV in past 12 months	48.3	24.7	4.6	7.7	−0.12(−0.17, −0.07)	<0.001

## Discussion

The findings of the quantitative research, supported by those of qualitative research published elsewhere [30], suggest that Zindagii Shoista had a very substantial impact on the lives of the families participating, particularly the younger women in the families. There was a marked improvement in socio-economic status, and reports of physical, sexual, and emotional IPV in the past year reported by women more or less halved, and this was supported generally by significantly lower reports of perpetration by men, although the latter reports may have been less reliable. The reports of a decline in IPV were supported by evidence of a significant decrease in depression among both women and men, more equitable gender attitudes, and perceived social norms in the community and less controlling behaviour towards women. These findings were supported by the qualitative research, which provided specific examples of changes in gendered practices and family relations [30].

For the most part, the changes reported were sustained more or less across the period from baseline to 30 months, that is 15 months after the end of the project support. While there were some reductions in indicators of women’s earnings and savings between 15 and 30 months, the positive results of the intervention largely were broadly sustained over the period from 15 to 30 months. There was some backsliding on some of the measures of relations between the mother-in-law and daughter-in-law and husband and wife between 15 and 30 months, which resulted in the overall trend not showing significant change. These may have been real changes, outside the gaze of the project team, or there could be a weakness in the measures as they were investigator-designed for the survey, and it is possible that they had not been sufficiently tested and optimised. The changes across the more established measures were more consistent.

To our knowledge, our research is the one of very few evaluations of interventions to prevent violence against women ever conducted in Tajikistan, or in the wider Central Asian region. This intervention was lightly adapted for use in Nepal where it was named the Sammanit Jeevan. It was evaluated with a very similar mixed methods study design, and the results of the evaluation similarly show considerable evidence of success in enhancing gender relations and family harmony [31]. The findings show that the intervention in Nepal was promising, and the economic aspect was very successful after the first year, but there were some differences in the research methods and the findings in respect of gender relations were stronger in Tajikistan.

The main strengths of the evaluation lie in the fact the quantitative findings were triangulated with qualitative research as well as monitoring data, which include compilation of a case study of each family. The findings of all data were supportive, and, in many respects, the qualitative data provided the most powerful examples of change. There was comparatively little loss of participants from the quantitative research cohort across the time points, and standard measures were used for each assessment. The project approach and methodologies were discussed and agreed among local and international project partners, and the local context was reflected in the project methodologies, drawing on the findings of the formative research [8], and included a range of measures developed for the study, particularly around mother-in-law and daughter-in-law relations. The limitations of the research include the small sample size and the lack of a control arm, which prevented comparison of families who had and had not had the intervention. Labour migration posed a major challenge as it meant that young men were not all around all the time during the intervention and research. However, we have greater confidence in the findings given that we have four data points, and all the findings were triangulated.

## Conclusion

The Zindagii Shoista intervention is one of the first evidence-based interventions to prevent violence against women in Tajikistan, and to our knowledge, the first to address the overlapping problems of poverty, patriarchy, and violence against women through a family focus. The research findings together show that the intervention approach shows particular promise in reducing IPV and other domestic violence against young women while simultaneously improving livelihoods, family dynamics, and emotional well-being. There is now evidence to support its promise from both Nepal and Tajikistan, which shows clearly that further research is warranted to assess the intervention’s effectiveness.

## Paper context

In Tajikistan, the high prevalence of violence against women and girls (VAWG) is driven by the nexus of patriarchy and poverty, as they intersect in multi-generational family units. We describe an evaluation of Zindagii Shoista, the first theoretically grounded, multi-generational approach to prevent VAWG in Tajikistan. Economic empowerment and gender-transformative approach showed a strong and sustained impact on gender relations and economic indicators. Further implementation and evaluation are indicated in Tajikistan and similar settings.
